# Persistent Cerebrospinal Fluid Leukocytosis: Could this be Idiopathic Intracranial Hypertension?

**DOI:** 10.7759/cureus.4607

**Published:** 2019-05-07

**Authors:** Waseem T Malik, Rubab Ali, Aun R Shah

**Affiliations:** 1 Neurology, Shifa International Hospital, Islamabad, PAK; 2 Surgery, Shifa International Hospital, Islamabad, PAK; 3 Internal Medicine, Case Western Reserve University School of Medicine / Metrohealth Medical Center, Cleveland, USA

**Keywords:** idiopathic intracranial hypertension, white cell count, cerebrospinal fluid, intracranial pressure, optic disc edema

## Abstract

A 37-year-old female, a known epileptic, presented to the neurology clinic with a seven-day history of persistent bilateral headache not improving with analgesics. Her neurological and systemic examinations were unremarkable except for right optic disc edema. Magnetic resonance imaging (MRI) brain and magnetic resonance venography (MRV) were normal but her cerebrospinal fluid (CSF) opening pressure was 280 mm of water with a CSF white cell count of 214. The patient showed improvement following treatment with intravenous antibiotics and acyclovir. She returned a week later with double vision and blurring in both eyes. Examination showed bilateral sixth nerve palsies and bilateral optic disc edema with left fundal hemorrhages. The spinal tap was repeated again, which showed a CSF opening pressure of 500 mm of water and the white cell count was 48. Extensive investigations for etiologies were mostly unrevealing. The patient was started on acetazolamide and topiramate combined with a large-volume therapeutic CSF tap. She continued to improve subsequently and was at the baseline functional state at three months, with complete resolution of hemorrhages and optic disc edema. Idiopathic intracranial hypertension (IIH) may present with persistent abnormal CSF with a high white cell count. Therefore, this must be diagnosed with caution, as it may be misdiagnosed and wrongly treated for other causes.

## Introduction

Idiopathic intracranial hypertension (IIH) is predominantly seen in young, obese females and characterized by headache, visual blurring, and tinnitus with bilateral papilloedema on examination [1]. Its annual incidence is 1-2/10000; whereas in obese females, its incidence is increased to 4-21/100000 [2]. Although IIH is most commonly idiopathic; it may be associated with a number of medical conditions, including chronic dural sinus thrombosis, and connective tissue diseases like neurosarcoidosis, systemic lupus erythematosus (SLE), and so on [3].

There are many other causes of intracranial hypertension, including drugs (such as lithium, vitamin A derivatives, oral or intrathecal steroids, nitrofurantoin, phenytoin, sulpha drugs, tetracyclines, and growth hormone treatments), hereditary conditions, and vitamin deficiencies or excesses [3]. Idiopathic intracranial hypertension is considered a diagnosis of exclusion by most authorities and it is paramount not to miss other etiologies [4]. These usually require completely different management strategies and, if left untreated, may lead to serious complications. Idiopathic intracranial hypertension is diagnosed by the modified Dandy criteria, which encompass signs and symptoms of raised intracranial pressure (ICP), including transient visual obscuration with headache and the presence of bilateral papilloedema on examination [5]. Cerebrospinal fluid (CSF) should have a normal composition with raised CSF pressure and unremarkable brain imaging, including magnetic resonance imaging (MRI) and venography. Most importantly, there should not be any other cause of raised intracranial pressure [6].

## Case presentation

A 37-year old female presented to the neurology clinic with a seven-day history of diffuse, persistent, and bilateral headache not improving with analgesics. There was no history of fever, loss of consciousness, blurring of vision, or gait abnormality. The patient was a known epileptic and was compliant with her anti-epileptic medication. On examination, her Glasgow Coma Scale (GCS) was 15/15, with normal speech and comprehension. She was afebrile and hemodynamically stable. Her cranial nerves examination was normal, except for right optic disc edema. The sensorimotor and systemic examination was unremarkable.

**Figure 1:**
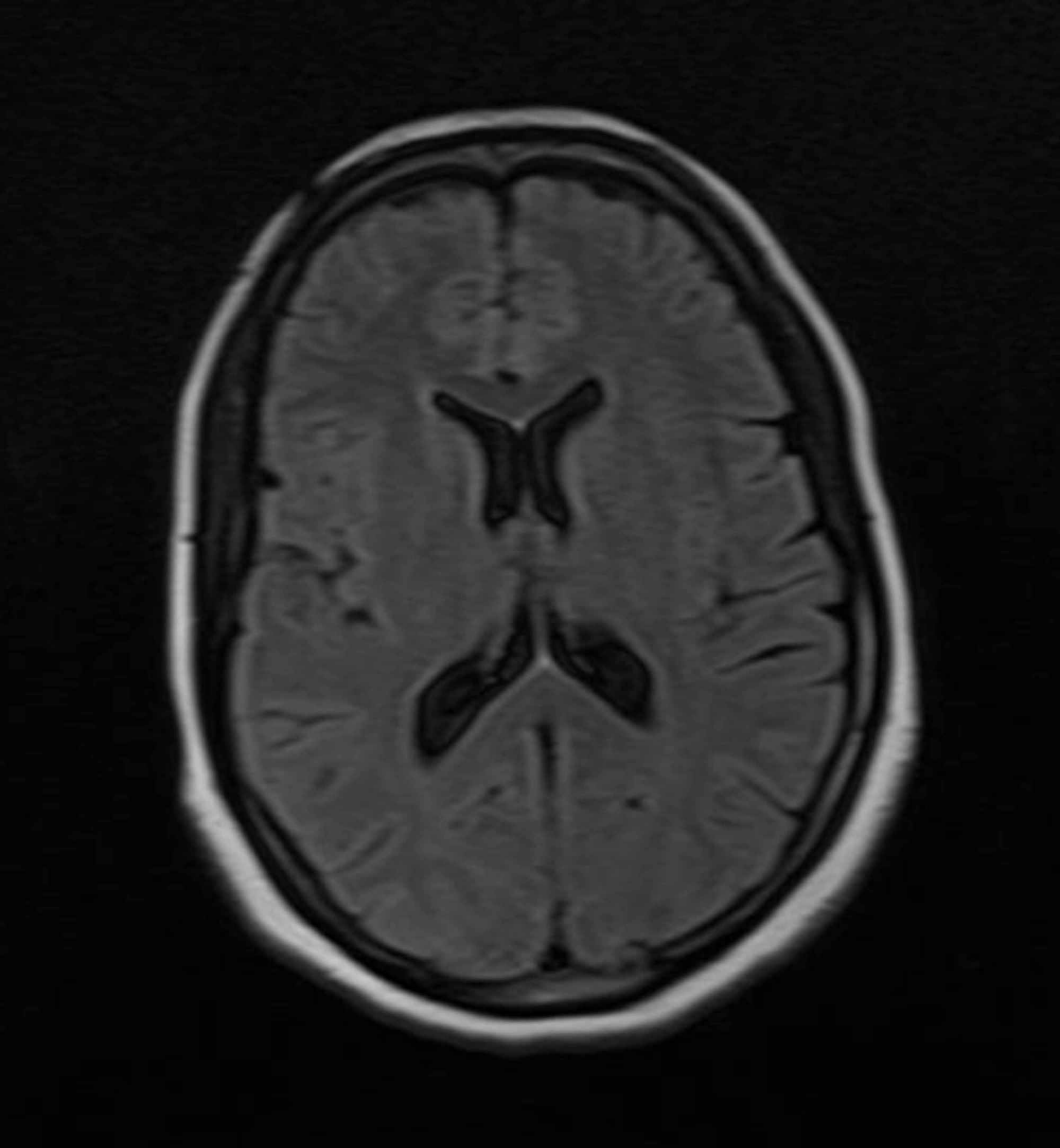
Non-contrast MRI brain (axial view) MRI = Magnetic Resonance Imaging

**Figure 2:**
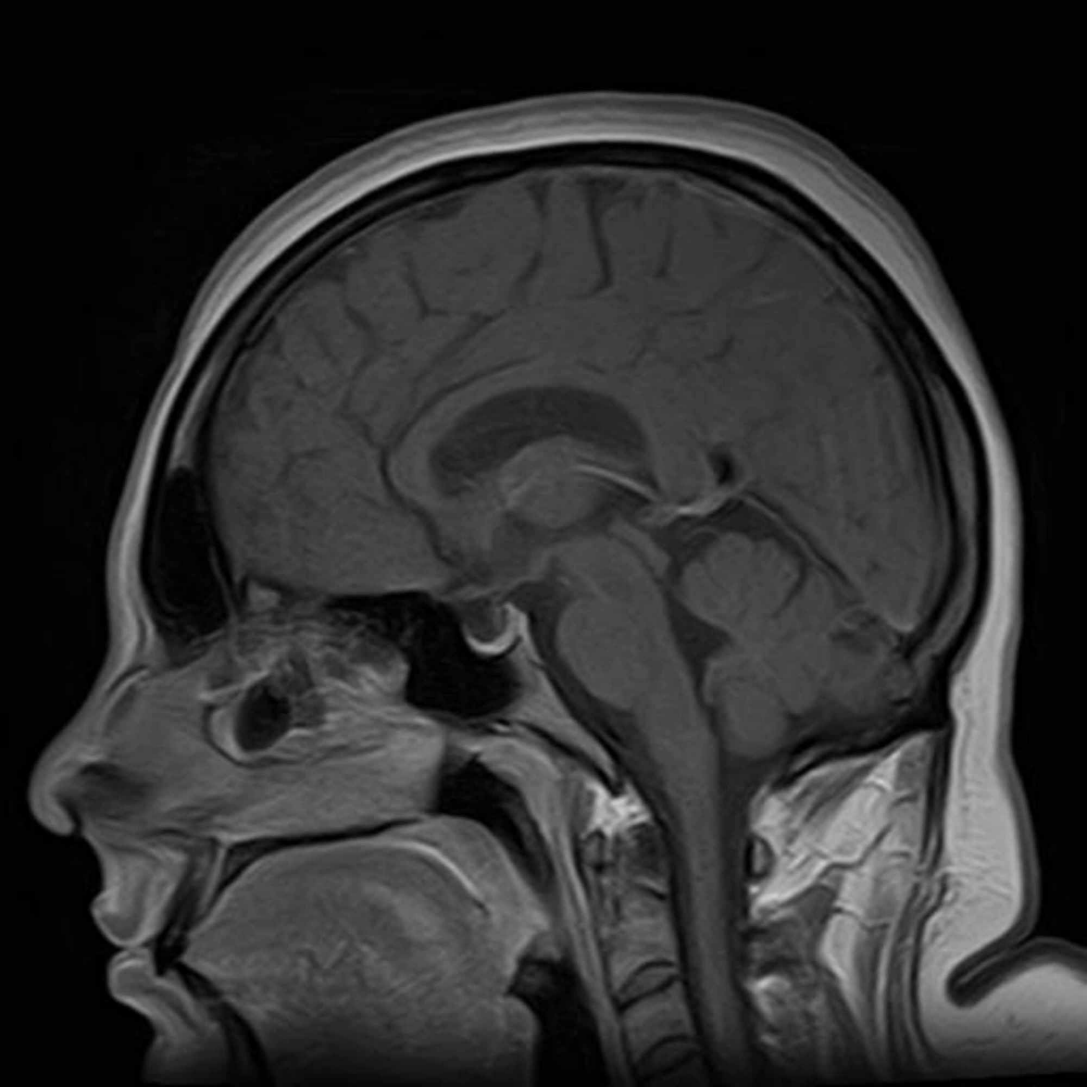
MRI brain with contrast (sagittal view) MRI = Magnetic Resonance Imaging

**Figure 3:**
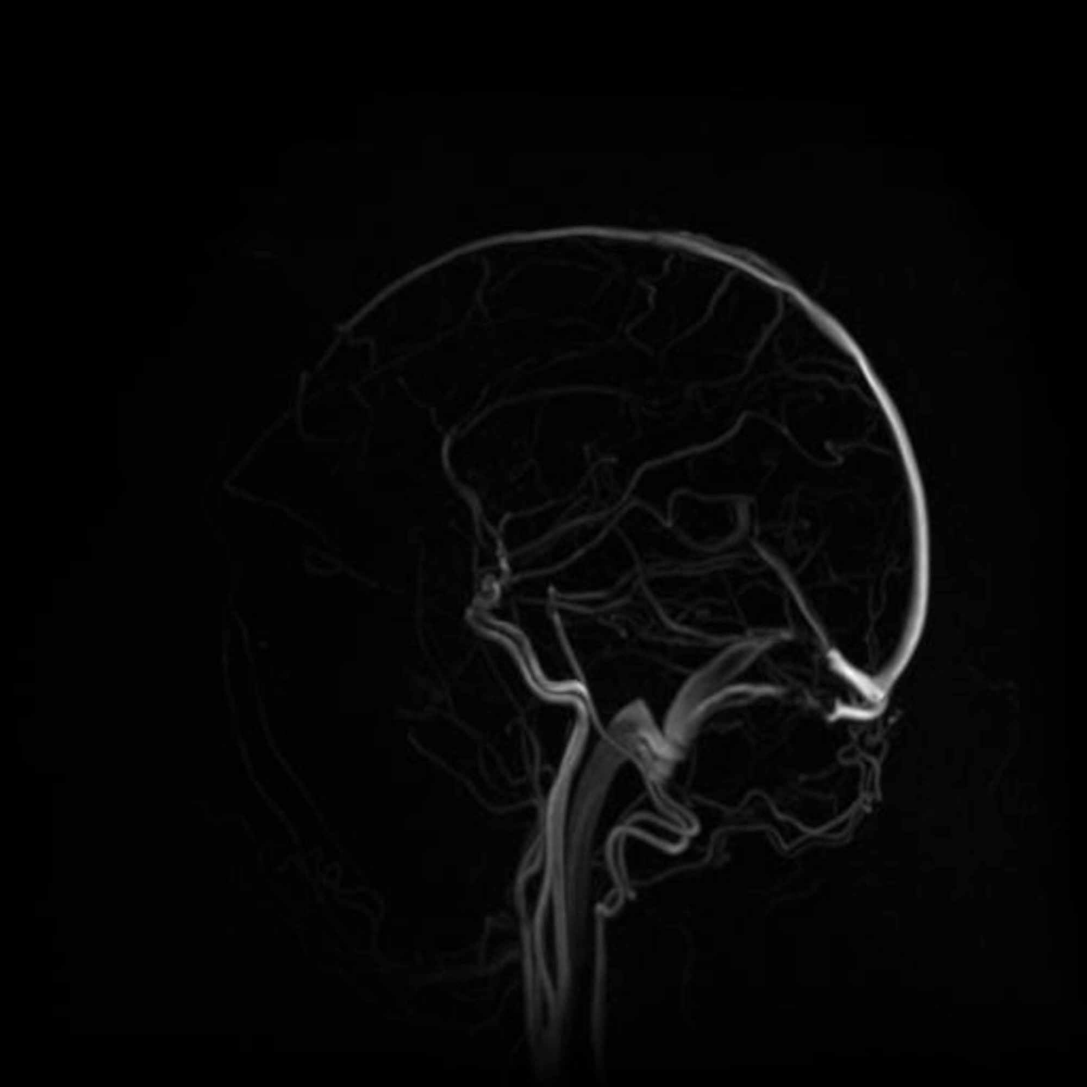
MRV brain showing normal flow (sagittal view) MRV = Magnetic Resonance Venography

**Figure 4:**
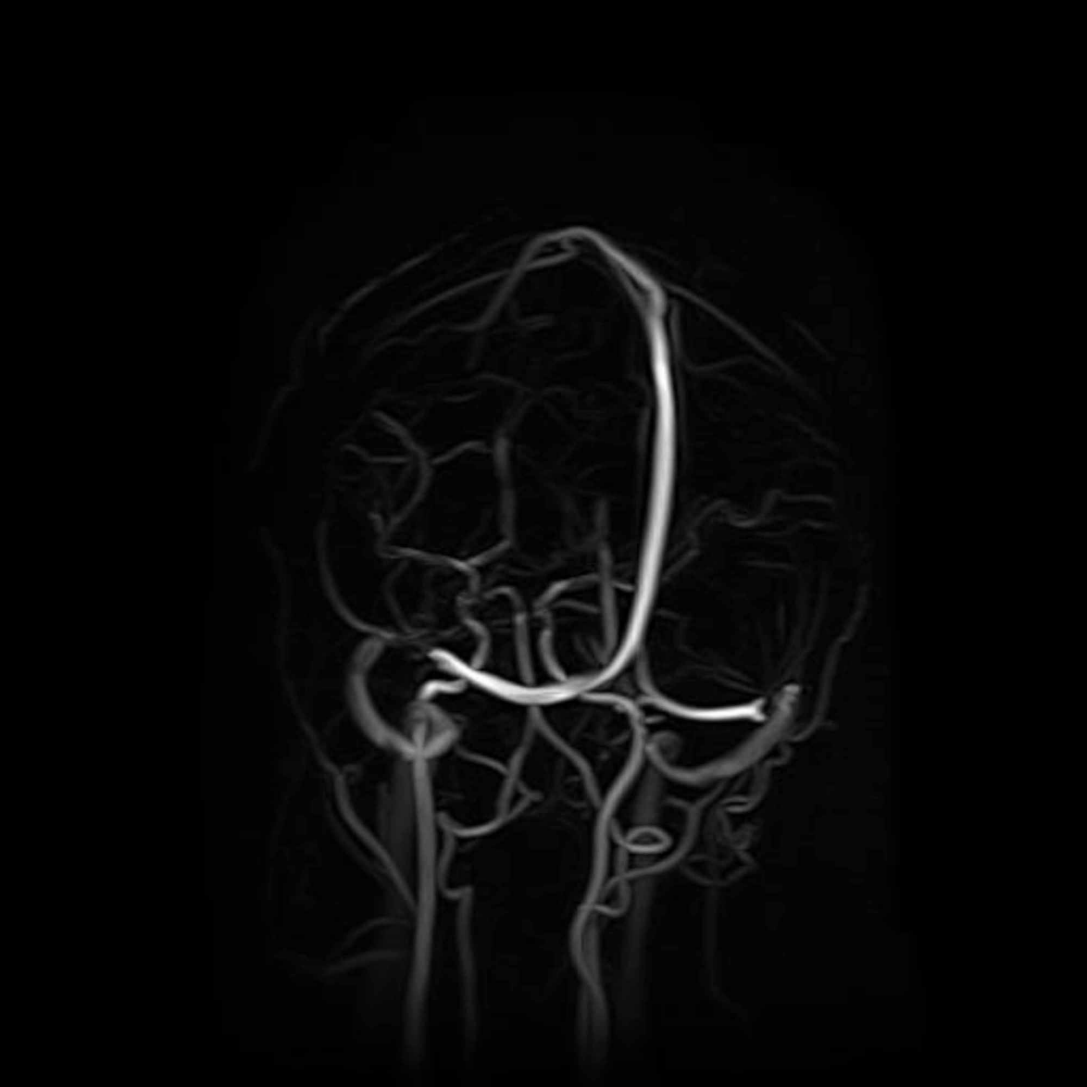
MRV brain (coronal view) MRV = Magnetic Resonance Venography

The patient was admitted to Shifa International Hospital, Islamabad, for further workup and management. Magnetic resonance imaging (MRI) of the brain (Figures 1-2) and magnetic resonance venography (MRV) (Figures 3-4) were normal.

Lumbar puncture revealed a CSF opening pressure of 280 mm of water and a white cell count of 241, with 90% lymphocytes and 10% neutrophils. CSF proteins and glucose were only mildly deranged (Table 1). All baseline investigations, including complete blood counts (CBC), erythrocyte sedimentation rate (ESR), and serum electrolytes, were unremarkable. Extensive testing for etiologies such as human immunodeficiency virus (HIV) serology, rapid plasma reagin (RPR), Treponema pallidum haemagglutination (TPHA), anti-neutrophil antibody (ANA) profile, and thyroid stimulating hormone (TSH) and serum angiotensin converting enzyme (ACE) levels did not unveil a causative pathology.

**Table 1:** Cerebrospinal fluid analysis comparison CSF = Cerebrospinal Fluid; WBC = White Blood Cells; RBC = Red Blood Cells; LDH = Lactate Dehydrogenase Date format: MM/DD/YY

CSF Indices (Reference Range)	11/17/17	11/30/17	12/12/17	12/21/17
Opening pressure (90-180 mmH_2_O)	280	500	40	200
WBC (0-5/mm^3^)	214	48	180	73
Neutrophils (approximately 0%)	5	10	5	20
Lymphocytes (approximately 70%)	95	90	10	80
RBC (0-10/mm^3)^	30	15	100	15
Glucose (45-80 mg/dL)	52	64	56	61
Protein (20-40 mg/dL)	66	22	58	39
LDH (<40 units/L)	<30	<30	<30	<30

The patient was started on intravenous (IV) antibiotics and IV acyclovir for a presumptive diagnosis of meningoencephalitis. She improved with treatment and was discharged home after five days on IV antibiotics and antiviral to complete the rest of the course at home.

**Figure 5:**
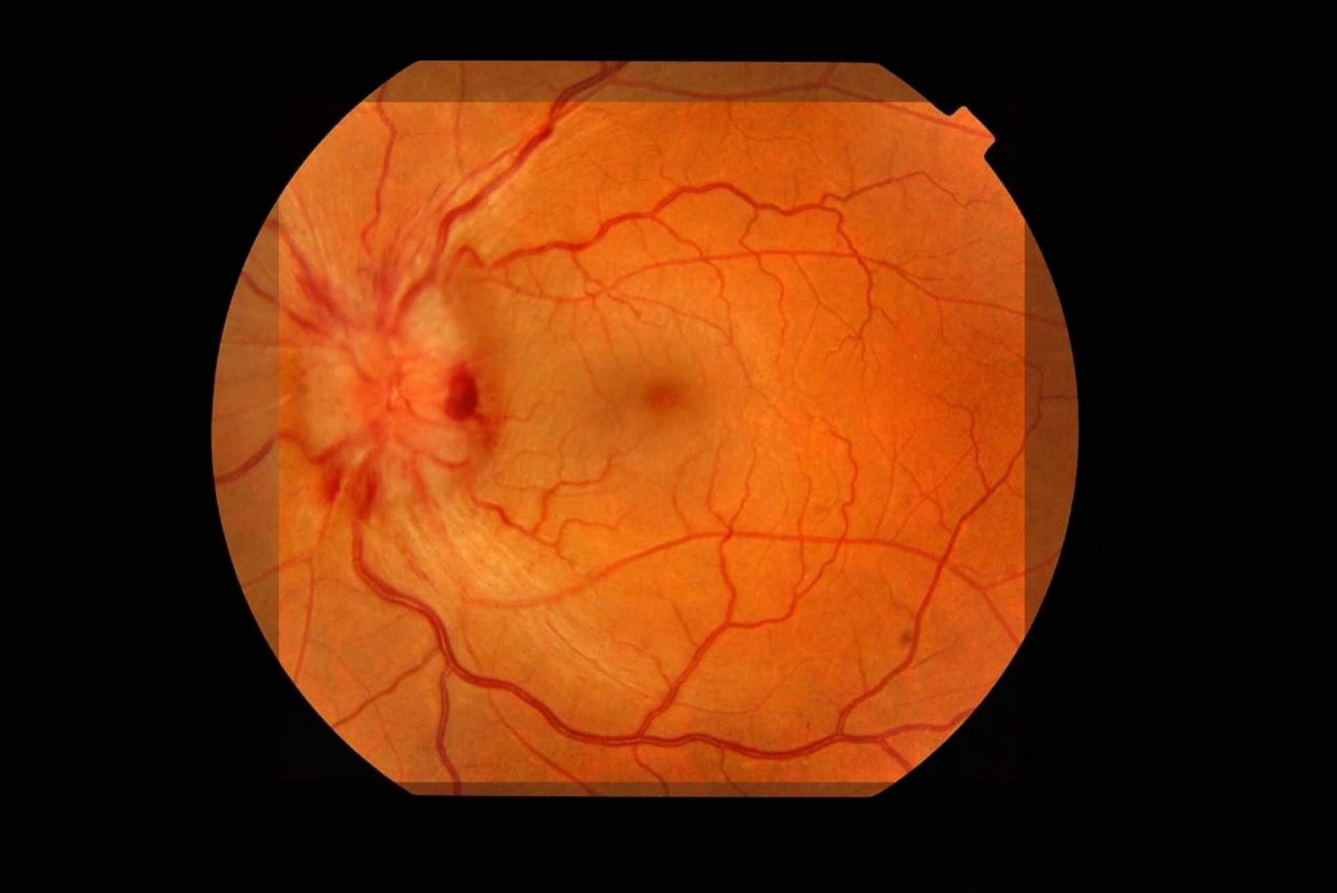
Left optic disc showing papilloedema and fundal hemorrhages

**Figure 6:**
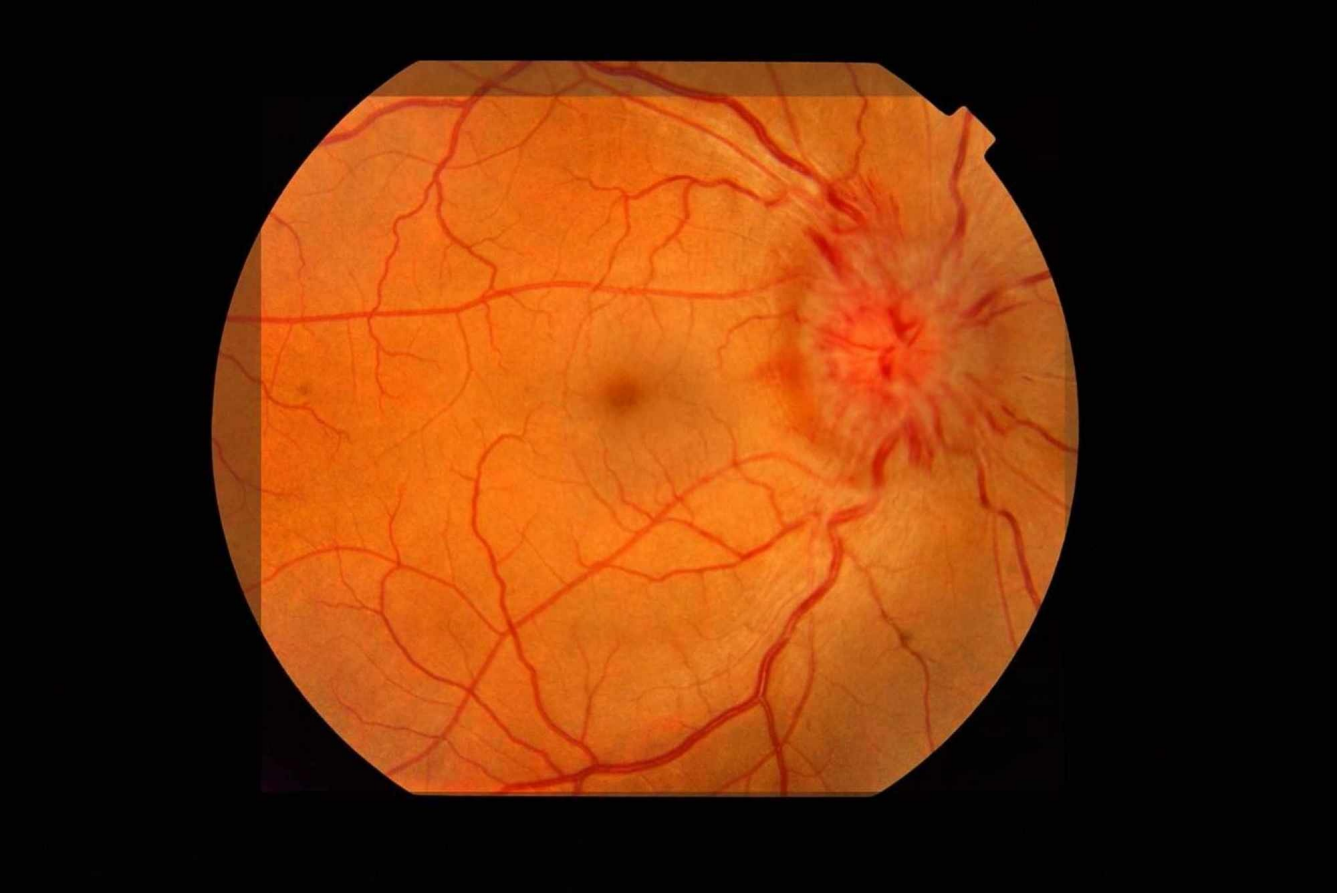
Right optic disc showing gross papilloedema

However, the patient returned after one week with double vision and blurring in both eyes. Examination revealed bilateral sixth nerve palsies and bilateral optic disc edema with left fundal hemorrhages (Figures 5-6). The rest of the neurological examination was normal. Systemic examination was unremarkable. Her spinal tap was repeated and CSF had an opening pressure of 500 mm of water and a white cell count of 48 with normal proteins and glucose. Thirty-five milliliters of CSF was drained, and the patient was started on oral acetazolamide and topiramate. The patient was also referred to neurosurgery for ventriculoperitoneal shunting but declined surgery.

On follow-up after 10 days, the patient manifested some improvement in vision and diplopia. On examination, her ocular movements were normal, however, with persistent bilateral optic disc swelling and left fundal hemorrhages. On this instance, the patient’s CSF pressure was 40 mm of water and white cell count of 95, predominantly lymphocytes (90%) with normal CSF glucose and proteins (Table 1). CSF gram staining, culture sensitivity, Mycobacterium tuberculosis polymerase chain reaction (MTB-PCR), serology for Cryptococcal antigen, and Indian ink staining for fungus were also negative.

She re-visited our outpatient clinic after nine days with static vision and considerable improvement in headache. On examination, her visual acuity was 6/12 in both eyes. Fundoscopy still showed bilateral papilloedema. The CSF examination was repeated and revealed an opening pressure of 200 mm of water and 78 white cells with normal proteins and glucose (Table 1). Doses of acetazolamide and topiramate were increased and she was sent home with advice to return in case of visual deterioration.

**Figure 7:**
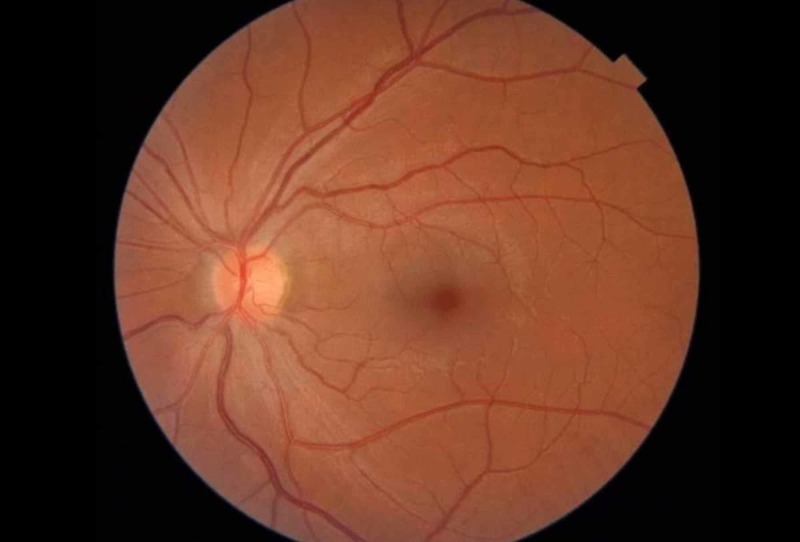
Left fundal photograph showing complete resolution of papilloedema

**Figure 8:**
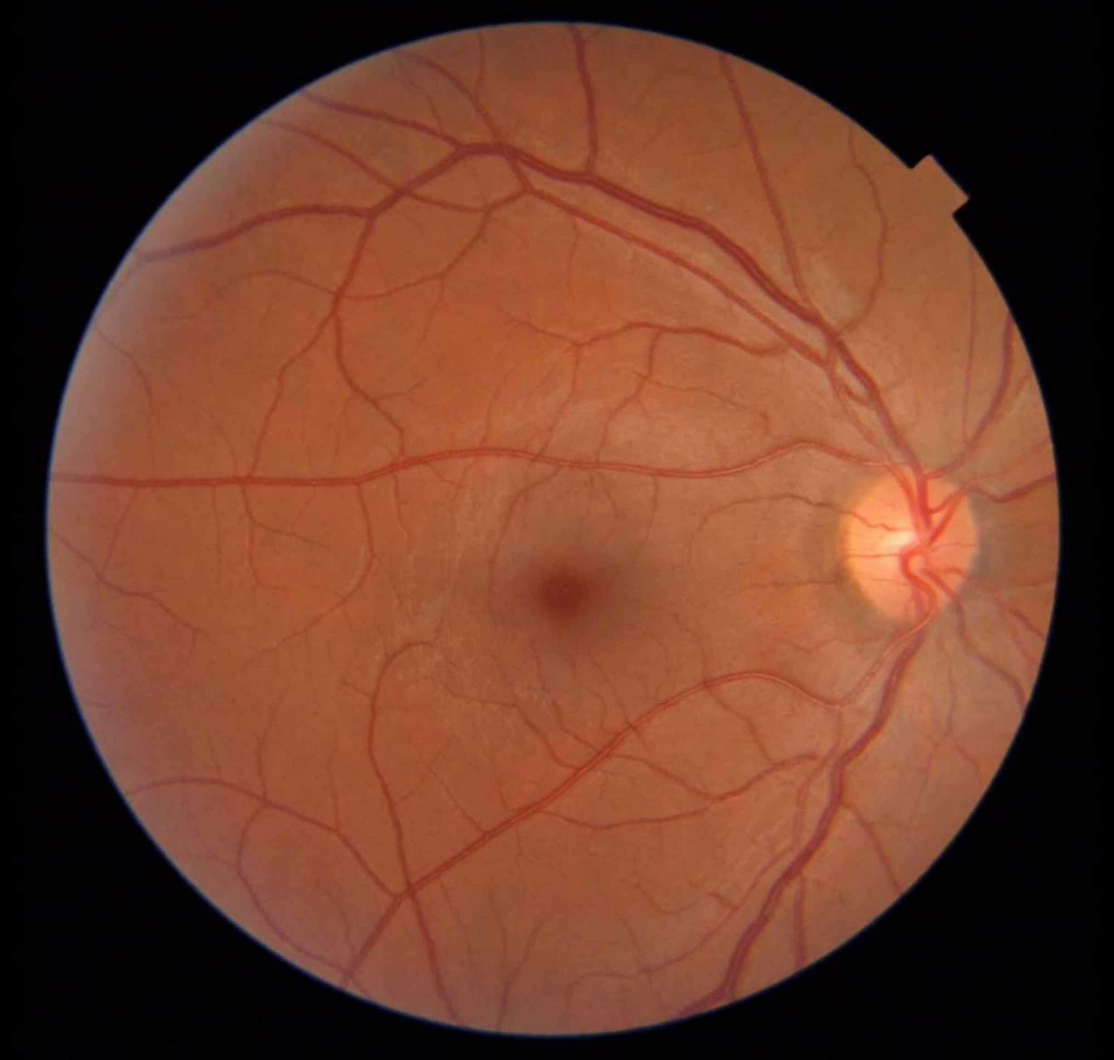
Right fundal photograph showing complete resolution of papilloedema

The patient’s progress was tracked telephonically over the next three months and she reported improved vision. At the three-month follow-up visit, her visual acuity was 6/6 and fundal examination showed the complete resolution of hemorrhages and optic disc edema (Figures 7-8).

## Discussion

Clinically and radiologically, our patient appeared to have idiopathic intracranial hypertension but her CSF findings were persistently abnormal, which makes diagnosis a challenge. There could be many possible differentials, including meningoencephalitis, and she was treated for that. There is a possibility of chronic meningitides like tuberculosis and fungal infections or rare causes like Lyme disease and Bechet’s disease. Inflammatory diseases of the brain such as neurosarcoidosis, systemic lupus erythematosus, Sjogren's syndrome, and vasculitis can also manifest in a similar manner [3,7]. Neoplastic infiltration, paraneoplastic encephalitis, and autoimmune encephalitis, like anti-NMDA (N-Methyl-D-aspartate) receptor encephalitis, can also have an analogous presentation [3].

Inflammatory diseases of the brain are sometimes difficult to diagnose in the absence of systemic diseases, however, in this case, these are very less likely given the subsequent improvement when treated only with acetazolamide [8]. One close differential is a transient headache and neurologic deficits with cerebrospinal fluid lymphocytosis (HaNDL) syndrome, which is characterized by migraine-like attacks with neurological deficits, papilloedema, CSF pleocytosis, and raised CSF pressure [9-10]. Other possible differentials include Mollaret meningitis, which is recurrent meningitis characterized by headache and CSF lymphocytosis but the disease course seen here was not recurrent; rather, it was one continuous process [11-12].

Our patient’s CSF polymerase chain reaction (PCR) for Mycobacterium tuberculosis and fungal organisms were negative. There were no malignant cells seen in the CSF. Neoplastic and inflammatory diseases were ruled out with the help of chest X-rays, ultrasound abdomen and pelvis, blood tests, including CBC, ESR, HIV serology, RPR, TSH, TPHA, ANA, and extractable nuclear antigen (ENA) antibody profile.

Despite extensive investigations for all possible causes of abnormal CSF, we could not find any other ongoing disease process to explain this presentation. Subsequently, her improvement with standard treatment for IIH could be considered a confirmation for this being IIH with abnormal CSF [7-8]. The literature search revealed five cases of IIH with abnormal CSF and the maximum number of cells reported was 152, whereas, in our patient, it was 241 [8]. Same as in our patient, these reported cases were initially also treated for presumed meningoencephalitis and investigated for autoimmune inflammatory diseases. This highlights the difficulty of establishing a diagnosis of IIH in the presence of persistently abnormal CSF findings. Investigating alternate diagnoses can prove to be time-consuming and expensive as well. Nonetheless, it is still paramount to exclude differentials whilst also being mindful of the fact that prompt treatment of IIH is necessary to prevent complications such as permanent visual impairment [3,5].

## Conclusions

Idiopathic intracranial hypertension can present with persistently abnormal CSF and high white cell count. Although this is extremely rare, one should be mindful of such a possibility. This should not, however, discourage from directing all efforts to seek alternative etiologies.
